# *Nfkb1* Inhibits LPS-Induced IFN-β and IL-12 p40 Production in Macrophages by Distinct Mechanisms

**DOI:** 10.1371/journal.pone.0032811

**Published:** 2012-03-12

**Authors:** Xixing Zhao, Erik J. Ross, Yanyan Wang, Bruce H. Horwitz

**Affiliations:** 1 Department of Pathology, Brigham and Women's Hospital, Boston, Massachusetts, United States of America; 2 Division of Emergency Medicine, Children's Hospital, Boston, Massachusetts, United States of America; University of Pecs Medical School, Hungary

## Abstract

**Background:**

*Nfkb1*-deficient murine macrophages express higher levels of IFN-β and IL-12 p40 following LPS stimulation than control macrophages, but the molecular basis for this phenomenon has not been completely defined. *Nfkb1* encodes several gene products including the NF-κB subunit p50 and its precursor p105. p50 is derived from the N-terminal of 105, and p50 homodimers can exhibit suppressive activity when overexpressed. The C-terminal region of p105 is necessary for LPS-induced ERK activation and it has been suggested that ERK activity inhibits both IFN-β and IL-12 p40 following LPS stimulation. However, the contributions of p50 and the C-terminal domain of p105 in regulating endogenous IFN-β(*Ifnb*) and IL-12 p40 (*Il12b*) gene expression in macrophages following LPS stimulation have not been directly compared.

**Methodology/Principal Findings:**

We have used recombinant retroviruses to express p105, p50, and the C-terminal domain of p105 (p105ΔN) in *Nfkb1*-deficient murine bone marrow-derived macrophages at near endogenous levels. We found that both p50 and p105ΔN inhibited expression of *Ifnb*, and that inhibition of *Ifnb* by p105ΔN depended on ERK activation, because a mutant of p105ΔN (p105ΔNS930A) that lacks a key serine necessary to support ERK activation failed to inhibit. In contrast, only p105ΔN but not p50 inhibited *Il12b* expression. Surprisingly, p105ΔNS930A retained inhibitory activity for *Il12b*, indicating that ERK activation was not necessary for inhibition. The differential effects of p105ΔNS930A on *Ifnb* and *Il12b* expression inversely correlated with the function of one of its binding partners, c-Rel. This raised the possibility that p105ΔNS930A influences gene expression by interfering with the function of c-Rel.

**Conclusions:**

These results demonstrate that *Nfkb1* exhibits multiple gene-specific inhibitory functions following TLR stimulation of murine macrophages.

## Introduction

The transcription factor NF-κB plays a central role in regulating innate immune gene expression [1]. While NF-κB is generally considered a pro-inflammatory factor, LPS induces significantly higher levels of the genes for IFN-β (*Ifnb*) and IL-12 p40 (*Il12b*) in macrophages derived from mice with targeted deletions in *Nfkb1*, the gene coding for the p50 subunit of NF-κB, than in WT macrophages [2]–[8]. Thus *Nfkb1* is an inhibitor of key immunoregulatory genes, and this is manifest in the observation that *Nfkb1^−/−^* mice are highly susceptible to microflora-induced colitis [9]. However, the molecular basis for selective inhibition of *Ifnb* and *Il12b* by *Nfkb1* has not been completely defined.

*Nfkb1* has multiple gene products. Its full-length gene product p105 is a bipartite molecule consisting of an N-terminal Rel homology domain and a C-terminal ankyrin repeat domain [10]–[15]. Constitutive processing of p105 by the proteosome removes the C-terminal domain of p105 to generate p50 monomers, which then homodimerize or heterodimerize with other NF-κB family members [16]. In contrast to the constitutive processing of p105 to p50, LPS induces IκB kinase (IKK)-dependent complete degradation of p105 [17]–[19]. IKK mediates the phosphorylation of p105 at serine 930, which is located within a C-terminal amino acid sequence partially homologous to the N-terminal IKK-dependent phospho-acceptor sites in IκB-α, and mutations of S930 inhibit IKK-mediated inducible degradation of p105 [18], [20], [21].

It has previously been suggested that p50 homodimers are inhibitory based on their ability to prevent binding of more active NF-κB dimers to κB sites necessary for inflammatory gene expression [22], [23]. Nevertheless, this does not explain why the absence of *Nfkb1* selectively affects expression of *Ifnb* and *Il12b*. Recently, it has been proposed that p50 binds to a G-rich sequence within the *Ifnb* interferon stimulated response element (ISRE) and interferes with recruitment of IRF-3. This potentially explains the selective increase in IFN-dependent gene expression observed in *Nfkb1^−/−^* macrophages [3], although it is not yet clear whether expression of p50 alone is sufficient to inhibit *Ifnb* expression in *Nfkb1^−/−^* macrophages. Further, while expression of *Il12b* does not appear to be dependent on IRF-3 [24], it does contain an ISRE in its proximal promoter region [25], [26], but it has not yet been determined whether p50 is sufficient to inhibit LPS-induced *Il12b*.

In contrast to the suggestion that p50 is responsible for *Nfkb1*-mediated inhibition of *Ifnb*, we have proposed that the ability of the C-terminal region of p105 to facilitate ERK activation is responsible for inhibition of LPS-induced *Ifnb* expression [8]. ERK inhibitors enhance LPS-induced expression of *Ifnb* and *Il12b* in macrophages [27]–[29]. The C-terminal portion of p105 binds to and stabilizes Tpl-2, a MAP3K that is essential for ERK activation [17], [30], and as a result, LPS-induced ERK activation is blocked in *Nfkb1^−/−^* bone marrow-derived macrophages (BMDM) [19]. It has been suggested that the basis of ERK-mediated inhibition is its ability to enhance induction of c-Fos, which inhibits both *Ifnb* and *Il12b* expression [27]–[29]. We have demonstrated that expression of a C-terminal fragment of p105 that does not contain the Rel homology domain (p105ΔN) rescues LPS-induced ERK activation and inhibits LPS-induced *Ifnb* expression [8]. However, whether the ability of p105ΔN to inhibit *Ifnb* expression depends upon ERK activation and whether p105ΔN is able to inhibit LPS-induced *Il12b* expression has not been determined.

To further delineate the molecular basis for *Nfkb1*-mediated inhibition, we have directly compared the ability of p105, p50, and p105ΔN to inhibit LPS-induced expression of *Ifnb* and *Il12b* in *Nfkb1^−/−^* murine bone marrow-derived macrophages. In addition, we have used a mutant of p105ΔN (p105ΔNS930A) that is unable to support ERK activation, to determine whether the inhibitory function of p105ΔN depends on its ability to activate ERK.

## Results

**Figure 1:**
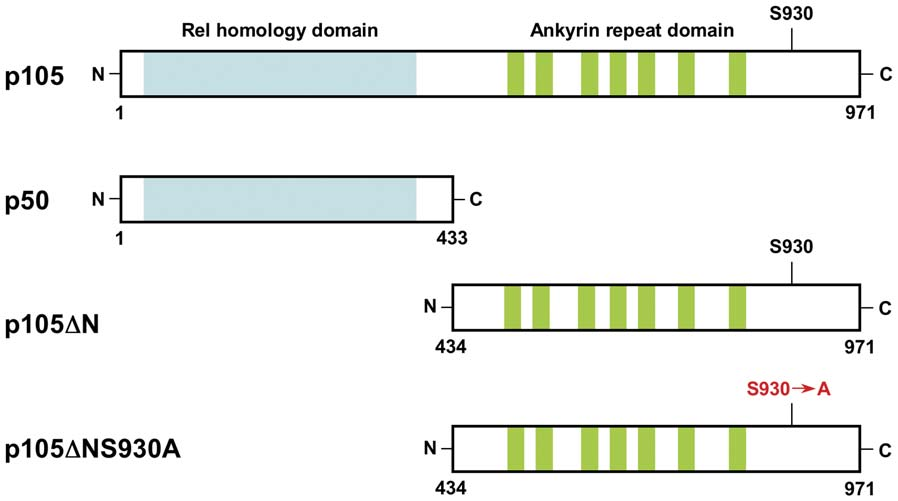
Domain structure of p105. Diagram showing p105, p50, and constructs p105ΔN and p105ΔNS930A.

**Figure 2:**
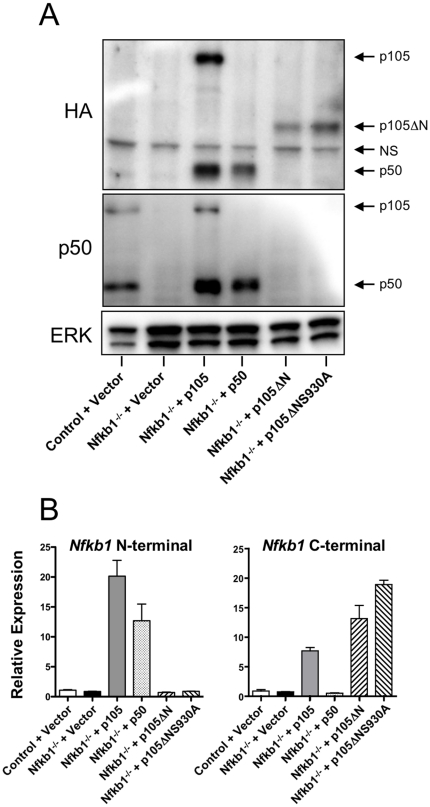
Retrovirally-mediated expression of *Nfkb1* constructs. A) Immunoblotting of *Il10* ^−/−^ *Rag2* ^−/−^ (Control) or *Nfkb1* ^−/−^ *Il10* ^−/−^ *Rag2* ^−/−^ (*Nfkb1* ^−/−^) BMDM infected with empty vector (Vector) or viruses expressing the indicated proteins. Total cell extracts from unstimulated cells were probed with the indicated antibodies (left) by western blotting. Arrows indicate size of *Nfkb1*-derived gene product (right). NS refers to a non-specific band. B) Relative expression of *Nfkb1* N- and C-terminal derived mRNA as determined by RT-PCR. N = 3 mice per group.

BMDM were infected with recombinant retroviruses that expressed either HA tagged full-length p105, p50 (amino acids 1–433), a C-terminal fragment of p105 which lacks the N-terminal Rel Homology Domain (p105ΔN, amino acids 434–971), or a mutant of p105ΔN in which S930, one of the serines in the C-terminal phosphorylation site necessary for IKK-dependent degradation and ERK activation, was mutated to alanine (p105ΔNS930A) [17], [20] (Fig. 1). Western blotting demonstrated expression of HA tagged proteins of appropriate sizes (Fig. 2A), and as expected we observed both p50 and p105 in macrophages infected with the vector expressing p105. Western blotting with an N-terminal p105 antibody indicated that levels of p50 and p105 in virally infected *Nfkb1* ^−/−^ cells were similar to endogenous levels observed in control cells (Fig. 2A). We observed lower protein levels of p105ΔN and p105ΔNS930A than p105, and it is likely that this is secondary to more rapid turnover, as we observed similar mRNA levels in BMDM expressing p105, p105ΔN, or p105ΔNS930A using a C-terminal *Nfkb1* Taqman RT-PCR probe (Fig. 2B, right). An N-terminal *Nfkb1* probe revealed similar RNA levels in cells expressing p105 and p50 (Fig. 2B, left).

**Figure 3:**
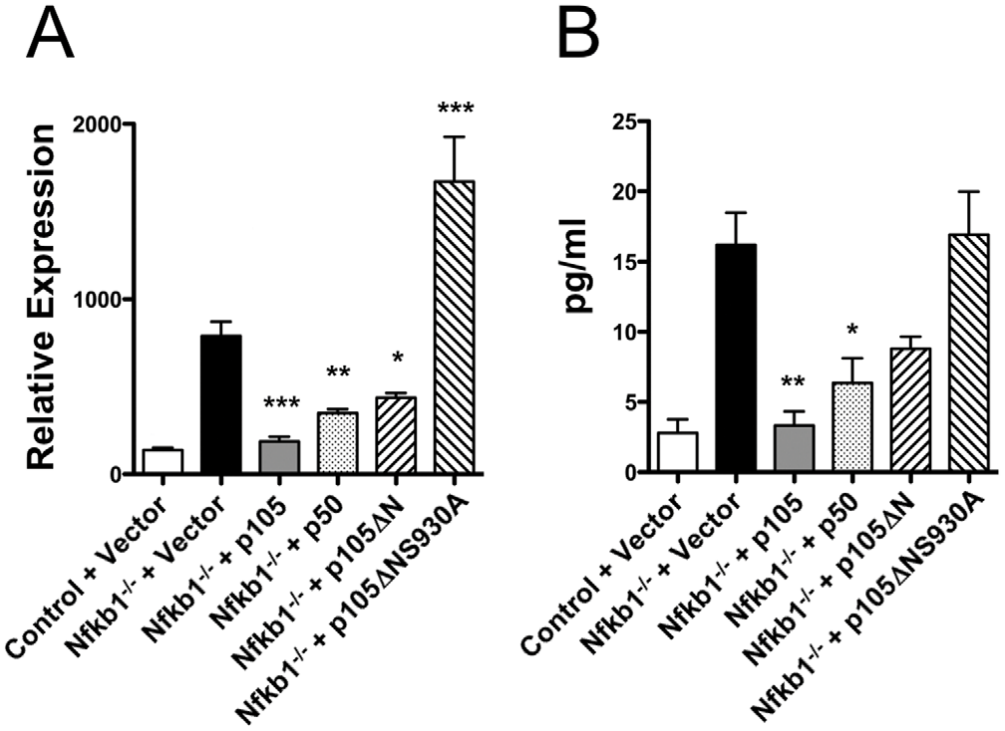
Both p50 and p105ΔN inhibit LPS-induced *Ifnb* in *Nfkb1* ^−/−^ macrophages. A) Relative expression of *Ifnb* as determined by RT-PCR in *Il10* ^−/−^ *Rag2* ^−/−^ (control) or *Nfkb1* ^−/−^ *Il10* ^−/−^ *Rag2* ^−/−^ (*Nfkb1* ^−/−^) BMDM infected with empty vector (Vector) or viruses expressing indicated proteins 2 hours after stimulation with LPS. N = 6–9 individual mice per group. B) Concentration of IFN-β in the supernatant of BMDM as determined by ELISA 12 hours after stimulation with LPS. N = 6 mice per group.

As expected, LPS induced significantly higher expression of *Ifnb* mRNA in *Nfkb1* ^−/−^ BMDM infected with vector alone than in control BMDM infected with the vector alone (Fig 3A). The presence of p105, p50, and p105ΔN in *Nfkb1* ^−/−^ BMDM all significantly inhibited expression of *Ifnb* mRNA at the point of peak induction 2 hours after LPS treatment, and these differences were mimicked by differences in IFN-β accumulation in the cell supernatants (Fig. 3B). These results indicate that both p50 and the C-terminal fragment of p105 independently inhibit expression of *Ifnb*.

**Figure 4:**
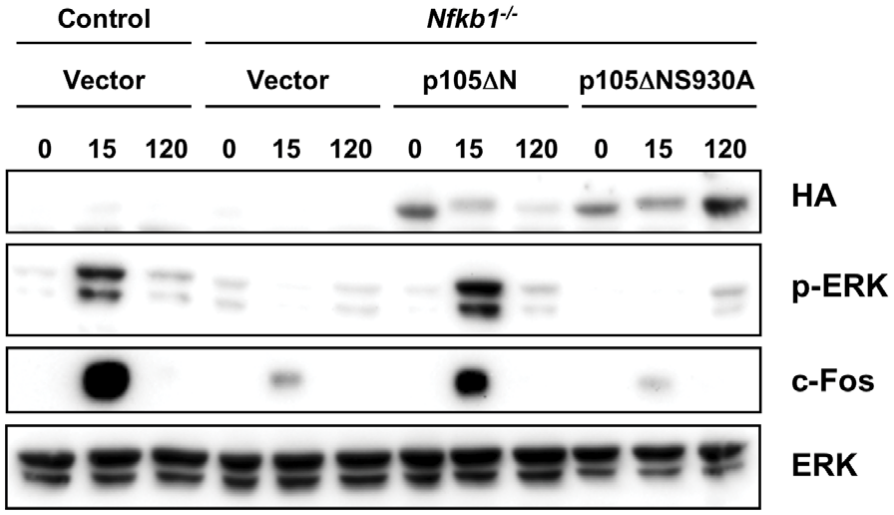
p105 serine 930 is essential for ERK activation and induction of c-Fos. Immunoblotting with indicated antibodies (right) of extracts derived from *Il10* ^−/−^ *Rag2* ^−/−^ (control) or *Nfkb1* ^−/−^ *Il10* ^−/−^ *Rag2* ^−/−^ (*Nfkb1* ^−/−^) BMDM infected with empty vector (Vector) or viruses expressing indicated proteins, prior to stimulation, or at 15 and 120 minutes after LPS stimulation.

It has previously been demonstrated that phosphorylation of S930 in p105 is essential for LPS-induced ERK activation [20], [21]. It has been proposed that ERK activation is associated with suppression of LPS-induced *Ifnb* and *Il12b* expression because it is necessary for induction of c-Fos, an inhibitor of both *Ifnb* and *Il12b* [29]. However, it has not been demonstrated whether phosphorylation at S930 is necessary for inhibition of *Ifnb* expression by p105ΔN. In contrast to p105ΔN, p105ΔNS930A did not inhibit LPS-induced *Ifnb* mRNA expression (Fig. 3A) or accumulation of IFN-β in cell supernatants (Fig. 3B), and in fact *Ifnb* mRNA was expressed at significantly higher levels in *Nfkb1* ^−/−^ BMDM expressing p105ΔNS930A than in *Nfkb1* ^−/−^ BMDM infected with vector alone (Fig. 3A). Consistent with a role for phosphorylation of serine 930 in LPS-induced ERK activation and stabilization of c-Fos, expression of p105ΔN but not p105ΔNS930A rescued the defect in ERK activation and c-Fos induction observed in *Nfkb1* ^−/−^ BMDM (Fig. 4). As expected, LPS-induced degradation of p105ΔNS930A was impaired (Fig. 4). These results demonstrate that phosphorylation of S930 plays an essential role in mediating the ability of the C-terminal region of p105 to inhibit LPS-induced *Ifnb* expression, and that this is strongly associated with activation of ERK and induction of c-Fos. Further, these results suggest that in the absence of IKK-mediated phosphorylation at S930, p105ΔN demonstrates an activity that enhances LPS-induced expression of *Ifnb* mRNA.

**Figure 5:**
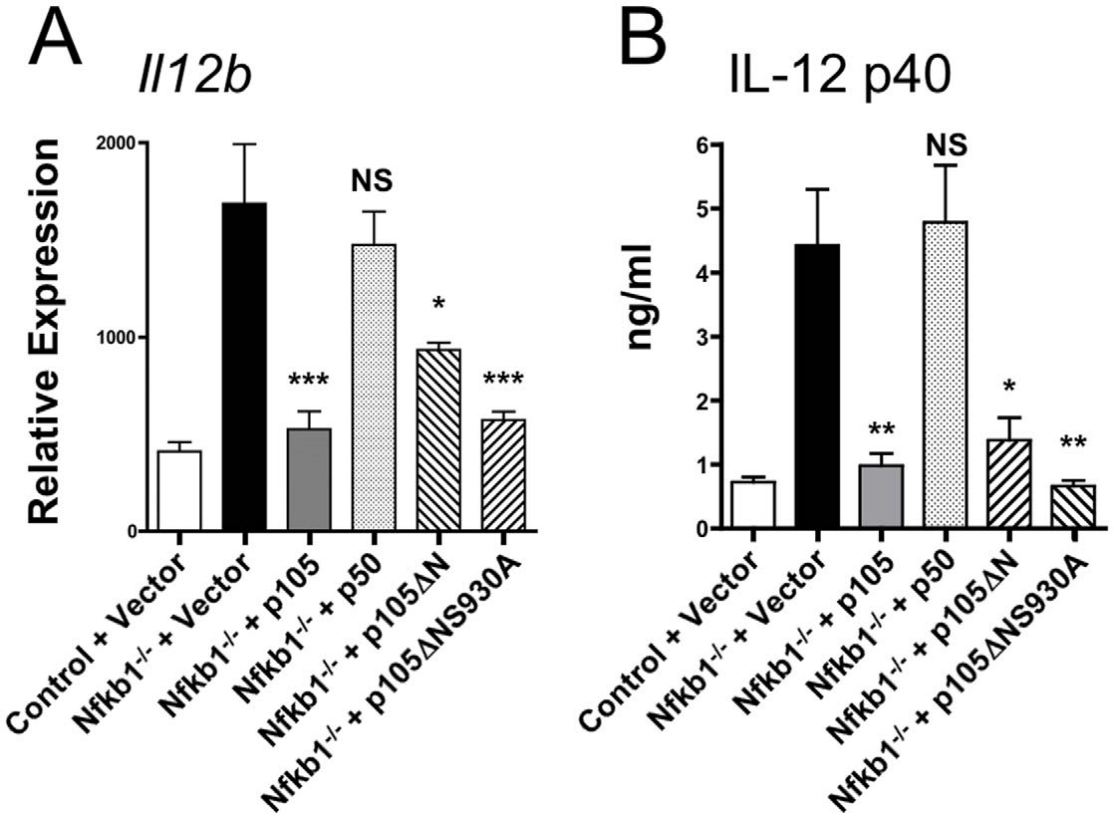
p105ΔN but not p50 inhibit LPS-induced *Il12b* expression in *Nfkb1* ^−/−^ macrophages. A) Relative expression of IL-12 p40 (*Il12b*) as determined by RT-PCR in *Il10* ^−/−^ *Rag2* ^−/−^ (control) or *Nfkb1* ^−/−^ *Il10* ^−/−^ *Rag2* ^−/−^ (*Nfkb1* ^−/−^) BMDM infected with empty vector (Vector) or viruses expressing the indicated proteins 4 hours after stimulation with LPS (N = 6–9 individual mice per group). B) Levels of IL-12 p40 present in the supernatant of groups described in A, 2 hours after stimulation with LPS (N = 3 individual mice per group).

Consistent with previous results [4], [6], [7], LPS also induced higher levels of *Il12b* in *Nfkb1* ^−/−^ cells than in control cells following infection with vector alone (Fig. 5). To determine whether *Nfkb1* inhibited *Il12b* expression using similar mechanisms employed for inhibition of *Ifnb*, we evaluated expression of *Il12b* in RNA samples from the virus-infected macrophages described above. As expected, p105 significantly inhibited expression of *Il12b* at both 2 (data not shown) and 4 hours after treatment with LPS (Fig. 5A). However, contrary to results with *Ifnb*, p50 did not significantly influence *Il12b* expression, suggesting that p50 is not an important inhibitor of *Il12b* following LPS treatment (Fig. 5A). In contrast, p105ΔN significantly inhibited expression of *Il12b*. However, we were surprised to find that inhibition did not depend on ERK activation or the induction of c-Fos, as p105ΔNS930A also significantly inhibited *Il12b* expression (Fig. 5A), demonstrating that inhibition of *Il12b* expression by p105ΔN was proceeding in an ERK and c-Fos independent fashion. Indeed, inhibition by p105ΔNS930A consistently appeared somewhat more robust than inhibition by p105ΔN, although this did not reach statistical significance. Differences observed in *Il12b* mRNA expression were closely correlated with differences observed in the accumulation of IL-12 p40 in cell supernatants (Fig. 5B).

**Figure 6:**
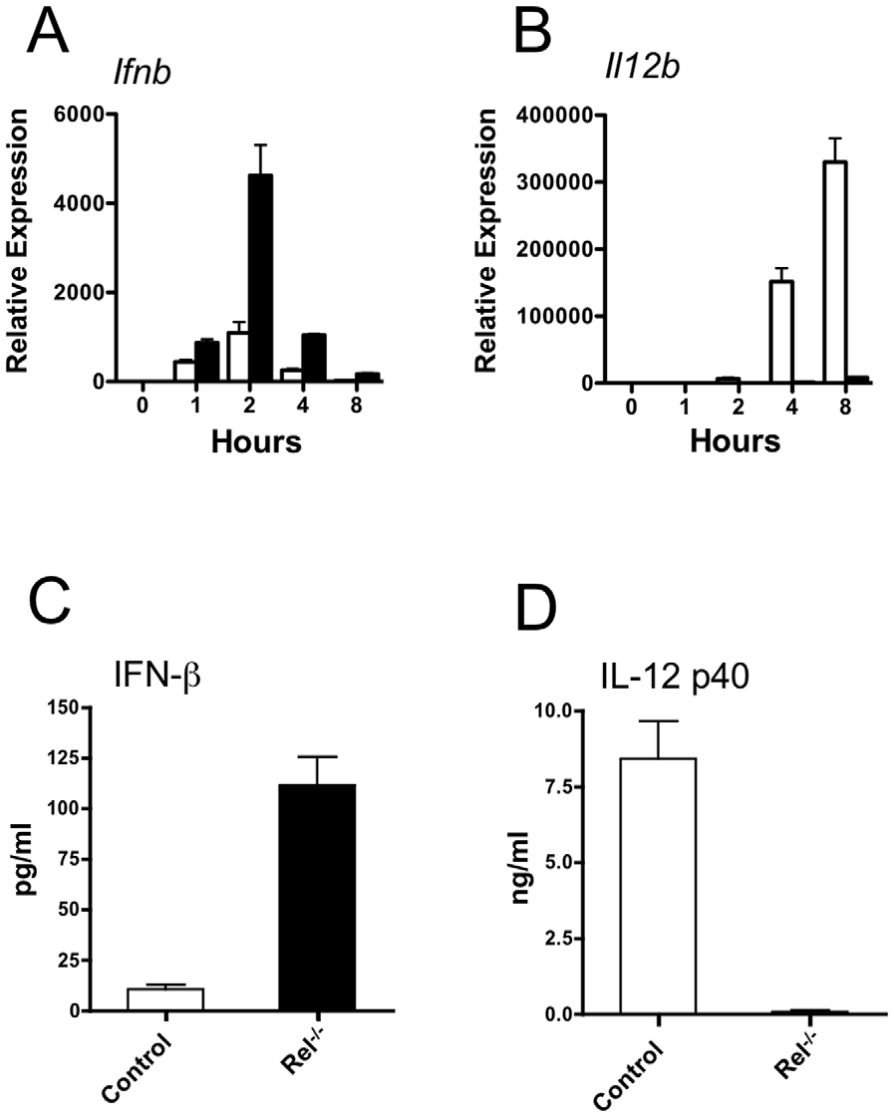
c-Rel inhibits LPS-induced expression of *Ifnb*. A) Relative expression of *Ifnb* and *Il12b* as determined by RT-PCR in *Il10* ^−/−^ *Rag2* ^−/−^ (open bars) and Rel^−/−^ *Il10* ^−/−^ *Rag2* ^−/−^ (filled bars) BMDM at indicated time points after stimulation with LPS. N = 3 mice per group. Repeated 3 times with similar results. B) Levels of IFN-β and IL-12 p40 in supernatants of *Il10* ^−/−^ *Rag2* ^−/−^ (Control) and Rel^−/−^ *Il10* ^−/−^ *Rag2* ^−/−^ (Rel^−/−^) BMDM 4 hours after stimulation with LPS. N = 5 mice per group.

The experiments described above employing p105ΔNS930A demonstrate that in the absence of the ability to activate ERK, the C-terminal region of p105 retains the ability to inhibit *Il12b* but enhances expression of *Ifnb* mRNA. In addition to facilitating LPS-induced ERK activation, the C-terminal region of p105 contains ankyrin repeats similar to those found in the classical IκBs [14], [15]. It has been shown that this region mediates association between p105 and both p65 and c-Rel [12], [15], [31], [32]. c-Rel is necessary for LPS-induced *Il12b* expression in macrophages [33], [34] and therefore inhibition of c-Rel function by the C-terminal region of p105 could explain the ability of p105ΔNS930A to inhibit *Il12b*. Conversely, inhibition of c-Rel function could explain the ability of p105ΔNS930A to augment *Ifnb* induction if c-Rel is an inhibitor of *Ifnb*. In fact, we observed that *Rel^−/−^* BMDM expressed significantly higher levels of *Ifnb* mRNA 2 hours after LPS stimulation than control macrophages, while, as expected, they expressed markedly lower levels of *Il12b* mRNA (Figs. 6A and 6B). These results were closely mimicked by the accumulation of IFN-β and IL-12 p40 in cell supernatants (Figs. 6C and 6D). These results suggest that inhibition of c-Rel function by the C-terminal region of p105 could have differential effects on the expression of *Il12b* and *Ifnb*.

## Discussion

We have shown that *Nfkb1* inhibits LPS-induced gene expression by multiple gene-specific mechanisms. p50 inhibits *Ifnb* but not *Il12b*. In contrast, the C-terminal region of p105 plays a role in inhibition of both *Ifnb* and *Il12b*, but only inhibition of *Ifnb* requires the ability of this C-terminal region to activate ERK.

It was previously proposed that homodimers of p50 inhibit access of more active Rel dimers to κB sites present with the promoter regions of NF-κB target genes [22]. However, a recent study demonstrated that promoters of genes expressed at higher levels in *Nfkb1*-deficient cells are enriched for ISRE-like sequences that contain short G-rich stretches [3]. It was proposed that these G-rich stretches serve as cryptic binding sites for p50 homodimers, which prevent recruitment of basal IRF-3. The promoter for *Ifnb* contains such an ISRE [3], but it has not been previously determined whether p50 alone is capable of inhibiting LPS-induced expression of endogenous *Ifnb* in *Nfkb1* ^−/−^ macrophages. Here we show that expression of p50 in *Nfkb1^−/−^* BMDM can inhibit expression of *Ifnb*, which is consistent with a model in which binding of p50 to the *Ifnb* ISRE inhibits *Ifnb* expression.

In contrast to its ability to inhibit *Ifnb* expression, p50 was unable to inhibit expression of *Il12b*. The fact that p50 was expressed above endogenous levels but was still unable to inhibit *Il12b* indicates that this failure was not an artifact of low expression levels. The *Il12b* promoter has previously been demonstrated to contain an atypical NF-κB binding site that consists of a stretch of Gs, termed an NF-κB half site [35], as well as an ISRE [25], [26]. However the presence of these sites is clearly not sufficient to support inhibition of *Il12b* by p50, indicating that p50 has highly selective inhibitory function. Whether this selectivity is solely based on the presence of G-rich ISRE sequences and inhibition of IRF-3 recruitment remains to be determined.

In contrast to p50, expression of p105ΔN inhibited LPS-induced expression of both *Ifnb* and *Il12b*. Previous studies have shown that ERK inhibitors increase LPS-induced expression of both of these genes [27]–[29], leading us, and others, to hypothesize that p105 inhibits by facilitating activation of ERK [29], [36]. It has been proposed that ERK-dependent inhibition is mediated by increased mRNA expression [29] and/or protein stabilization of c-Fos [27], an inhibitor of both *Ifnb* and *Il12b* [27]–[29]. The results reported here strongly suggest that the ability of p105ΔN to rescue ERK activation and induce c-Fos is necessary to inhibit *Ifnb*, but in contrast, is dispensable for inhibition of *Il12b*.

The C-terminal fragment of p105 contains multiple ankyrin repeats similar to those found in other IκBs [11], [12], [14], [15], and it has been suggested that p105 may preferentially interact with c-Rel [12], [15], [31], [32]. c-Rel is essential for LPS-induced expression of *Il12b* [33] and thus inhibition of c-Rel function by p105ΔN could explain inhibition of *Il12b*. This is supported by the observation that p105ΔNS930A, which is resistant to IKK-mediated degradation, may be a better inhibitor of *Il12b* expression than p105ΔN itself. Interestingly, we have found that in contrast to its effects on *Il12b* expression, c-Rel inhibits LPS-induced expression of *Ifnb*. Thus, more robust inhibition of c-Rel function by p105ΔNS930A than by p105ΔN could explain why p105ΔNS930A actually augments expression of *Ifnb*. While one potential mechanism for inhibition of c-Rel function by p105ΔNS930A is inhibition of nuclear localization following LPS stimulation, we have not observed increased c-Rel nuclear translocation in *Nfkb1*-deficient BMDM nor have we observed inhibition of c-Rel nuclear translocation by p105ΔNS930A (data not shown). Therefore the functional consequences of c-Rel interaction with p105 remain to be fully elucidated.

The studies presented here provide new insight into the mechanisms of gene inhibition by *Nfkb1*. They demonstrate that *Nfkb1* has multiple inhibitory mechanisms that function in gene-specific manners. Further they illustrate that rather than an on/off switch for inflammation, NF-κB is a complex modulator of the innate response that differentially influences key immunoregulatory cytokines. Understanding the molecular details of gene-specific regulation by NF-κB could lead to highly selective therapeutic modulation of immune and inflammatory based diseases.

## Materials and Methods

### Ethics Statement

This study was carried out in strict accordance with the recommendations in the Guide for the Care and Use of Laboratory Animals of the National Institutes of Health. The animal protocol was approved by the Harvard Medical Area Standing Committee on Animals (OLAW Assurance number: A34131-01). All efforts were made to minimize suffering.

### Experimental Animals

All mouse strains were maintained on the 129S6/SvEvTac background. *Il10* ^−/−^ *Rag2* ^−/−^ (control) and *Nfkb1* ^−/−^ *Il10* ^−/−^ *Rag2* ^−/−^ (*Nfkb1* ^−/−^) mice have been previously described [8]. *Rel^−/−^Il10* ^−/−^ *Rag2* ^−/−^ (Rel^−/−^) mice were generated by intercrossing *Rel^−/−^Rag2* ^−/−^ [8] and *Il10* ^−/−^ *Rag2* ^−/−^ mice. Macrophages were derived from mice maintained on the *Il10^−/−^* background to eliminate the effects of the strong IL-10 feedback loop on LPS-induced gene expression, and on the *Rag2^−/−^* background to prevent the development of spontaneous colitis.

### Retrovirally-mediated gene expression

BMDM were grown as previously described [36]. On the second day of culture cells were infected with the indicated retroviruses. 72 hours later macrophages were re-plated at 5×10^5^ cells/well in 0.5 ml of medium in a 24-well plate, or at 0.3–1×10^5^ cells/well in 100 µl of medium in a 96 well plate. The following day cells were stimulated with LPS from *E. coli* 0127:B8 (Sigma, St Louis, MO) at 1 ng/ml or left unstimulated.

### RT-PCR

RNA was isolated in Trizol (Invitrogen, Carlsbad, CA) following the manufacturer's instructions. RT-PCR analysis was performed using probes from Applied Biosystems (Foster City, CA), as per the manufacturer's instructions. Expression for each sample was analyzed in duplicate and normalized to GAPDH using the ΔΔCt method. Fold-change is reported as relative to the average of uninduced levels in unstimulated BMDM for each experiment.

### ELISA

IFN-β was analyzed in culture supernatants using an IFN-β-specific ELISA kit (PBL Biomedical Laboratories) according to the manufacturer's instructions. IL-12 p40 was analyzed via ELISA using C15.6 (Biolegend, San Diego, CA) as the capture antibody and C17.8 (Thermo Scientific, Rockford, IL) as the secondary antibody.

### Antibodies

Anti-ERK (p44/42 MAP Kinase), anti-phospho ERK (p44/42 MAP Kinase) (Thr202/Tyr204), and anti-c-Fos (9F6) were purchased from Cell Signaling (Beverly, MA). Anti-N-terminal p105 (sc-1190) was purchased from Santa Cruz Biotechnology (Santa Cruz, CA). Anti-HA (16B12) was purchased from Covance (Princeton, NJ).

### Statistical Analysis

All data analysis was performed using GraphPad Prism software (GraphPad Software, inc., San Diego, CA). Data generated by RT-PCR or ELISA was compared using One-way ANOVA with the Tukey's multiple comparison test. Error bars on graphs represent SEM. * indicates p<0.05, ** indicates p<0.01, and *** indicates p<0.001.
